# Probing the Effect of MWCNT Nanoinclusions on the Thermoelectric Performance of Cu_3_SbS_4_ Composites

**DOI:** 10.1021/acsomega.2c06823

**Published:** 2022-12-15

**Authors:** Vaskuri C. S. Theja, Vaithinathan Karthikeyan, Dani S. Assi, Saianand Gopalan, Vellaisamy A. L. Roy

**Affiliations:** † Department of Materials Science and Engineering, City University of Hong Kong, Kowloon Tong, Hong Kong; ‡ Department of Electronics and Nanoscale Engineering, James Watt School of Engineering, University of Glasgow, G12 8QQGlasgow, United Kingdom; § Global Centre for Environmental Remediation (GCER), College of Engineering, Science and Environment, The University of Newcastle, Callaghan2308, New South Wales, Australia

## Abstract

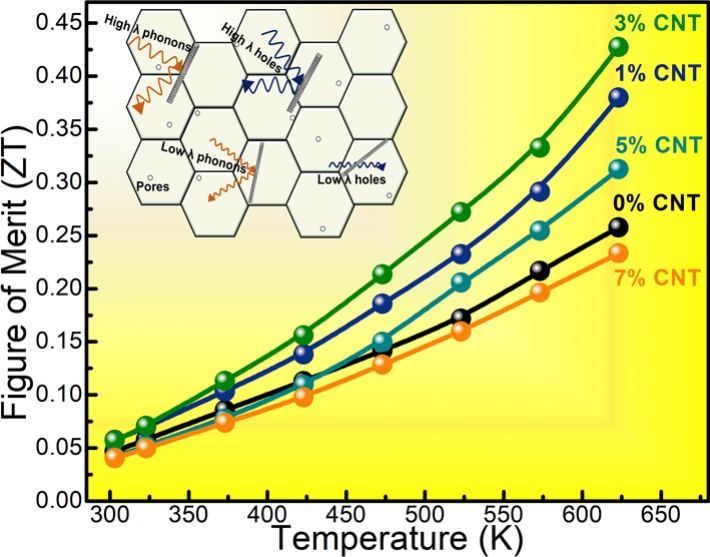

Recently, copper-based chalcogenides, especially sulfides, have attracted considerable attention due to their inexpensive, earth-abundance, nontoxicity, and good thermoelectric performance. Cu_3_SbS_4_ is one such kind with p-type conductivity and high phase stability for potential medium-temperature applications. In this article, the effect of a multiwalled carbon nanotube (MWCNT) on the thermoelectric parameters of Cu_3_SbS_4_ is studied. A facile synthesis route of mechanical alloying (MA), followed by hot pressing (HP) was utilized to achieve dense and fine-grain samples. Adding the optimal amount of MWCNT nanoinclusions in Cu_3_SbS_4_ enhanced the Seebeck coefficient by carrier energy filtering and reduced the thermal conductivity by strong phonon scattering mechanisms. This synergistic optimization helped achieve the maximum figure of merit (*ZT*) of 0.43 in the 3 mol % MWCNT nanoinclusion composite sample, which is 70% higher than the pristine Cu_3_SbS_4_ at 623 K. In addition, enhancement in mechanical stability is observed with the increasing nanoinclusion concentration. Dispersion strengthening and grain boundary hardening mechanisms help improve mechanical stability in the nanocomposite samples. Apart from the enhanced mechanical stability, our study highlights that the incorporation of multiwalled CNT nanoinclusions boosted the thermoelectric performance of Cu_3_SbS_4_, and the same strategy can be extended to other next-generation and conventional thermoelectric materials.

## Introduction

On-demand, global fossil fuels are nonrenewable, release CO_2_ (carbon dioxide) and GHG (greenhouse gas) pollutants, and produce more than two-third of energy in the form of waste heat.^1^ Alternative sustainable and renewable energy sources utilizing thermal energy to generate useful energy are the best solution for the continuously increasing energy demand, carbon neutrality, and attaining net-zero emissions.^2,3^ Currently, researchers are focused on solar, wind, geothermal, and thermoelectric renewable energy sources to improve their power conversion efficiencies. Thermoelectric power generation (TEGs) is an attractive eco-friendly, solid-state energy conversion system that directly converts waste heat into electricity.^4^ Commercial thermoelectric devices comprise toxic, expensive, rare-earth elements of Pb- and Te-based legs and apply to near-room-temperature applications.^5^ However, commercial TEGs work functioning diminishes if the applicable temperature is beyond room temperature.^3^ Statistical analysis suggested that 80% of industrial waste heat releases the temperature between 373 and 573 K.^6^ Passing over 200 years of research into thermoelectric materials, the scientific community still cannot find a solid alternative solution for traditional devices. Therefore, studying new thermoelectric materials made of inexpensive, earth-abundant, and nontoxic elements is a strong case of research interest for intermediate-temperature applications.

The heat of the electricity conversion efficiency of thermoelectric material is measured by its figure of merit (*ZT*), *ZT =* α^2^σ*T*/*κ*, where σ and κ are the electrical and thermal conductivities, α is the Seebeck coefficient, and T is the absolute temperature. Therefore, thermoelectric performance would be improved in two ways: by enhancing the power factor (α^2^σ) or suppressing the thermal conductivity.^7^ The lattice phonons and change carriers carry the heat; total thermal conductivity is a summation of phonon and electronic thermal conductivities. The big challenge in enhancing the power factor originates from the interrelation between carrier concentration and mobility.^8^ For example, it is evident that increasing charge carrier density improves the electrical conductivity (improves *ZT*), simultaneously reduces the Seebeck coefficient (reduces *ZT*), and increases thermal conductivity (reduces *ZT*).^9^ Therefore, it is clear that enhancing *ZT* by optimizing charge carrier density is quite challenging due to the complex interrelation between the electrical and thermal conductivities and the Seebeck coefficient.^10^ However, the formation of multiphase structures (forming nanocomposite) helps in decoupling the electron transport from phonon transport due to the presence of interfaces.^8^ Therefore, controlling the phonon transport by nanocomposite to reduce thermal conductivity is simple and results in improving *ZT* effectively.

Nanostructuring is grain boundary-based method and nanocompositing is an interfacial boundary-based method to improve the *ZT* by employing boundary-based phonon scattering to reduce lattice thermal conductivity.^4^ Nanostructuring produces fine-grain boundaries during synthesis like mechanical alloying and chemical synthesis, and compositing derives from adding secondary dispersoids like nanotubes and nanoparticles.^4^ In both methods, the primary target is to improve the TE performance through a boundary-based phonon scattering mechanism to reduce phonon thermal conductivity. The advantage of nanocompositing is maintaining or improving the power factor by enhancing the Seebeck coefficient by energy-dependent carrier filtering or electrical conductivity by improving the electrical density of the state’s mechanism. In most cases with nanocompositing, researchers frequently observed the carrier energy filtering due to the low-energy interfacial boundaries scattering.^4,11,12^ However, electrical conductivity enhancement was significantly unpredictable due to the reduction in carrier mobility from the interfacial boundary scattering.^13^ In addition, to improve thermoelectric performance, nanocompositing also enhances the mechanical properties, which are essential for devices’ operational durability and manufacturing process.^10^

In addition to high thermoelectric performance (*ZT*), earth-abundance, phase and mechanical stability, and nontoxicity are the other desired characteristics for developing large-scale commercialization.^14^ In this regard, Ge et al.^5^ statistically proved that sulfides are the best alternative and economical solution for traditional TE materials. Sulfides have other advantages like low cost, nontoxicity, and earth-abundance (in the Earth’s crust, sulfur is almost 1000 times more abundant than selenium).^15^ Ternary copper-based chalcogenides Cu–Sb–S are gaining significant attention as promising p-type materials due to their excellent TE performance.^16−25^ The Cu–Sb–S system has four possible TE phases at room temperature: famatinite (Cu_3_SbS_4_), chalcostibite (CuSbS_2_), skinnerite (Cu_3_SbS_3_), and tetrahedrite (Cu_12_Sb_4_S_13_). These phases are naturally occurring minerals in the Earth’s crust and excellent semiconducting TE phases.^26^ In another work, Mashadiyeva et al.^26^ thermodynamically proved that the Cu_3_SbS_4_ phase stability is higher than that of other Cu–Sb–S phases. The Earth-abundant ternary chalcogenide Cu_3_SbS_4_ is one of the potential p-type semiconductors with zinc blende (ZnS) derivative tetragonal (*I*4̅2*m*) structure.^14^ Cu_3_SbS_4_ has a band gap of around 0.5–1 eV with good TE performance for intermediate-temperature applications.^27^ Cu_3_SbS_4_ exhibits a relatively high thermal conductivity, mainly attributed to its low structural complexity associated to high phonon thermal conduction.^14^ This is possibly due to the feature of its small and uncomplicated primitive cell structure and the sulfur’s low atomic weight.^14^ Toward that end, Zhang et al.,^28^ incorporated the SiC nanoparticles in the Cu_3_SbS_4_, which successfully resulted in phonon scattering to reduce thermal conductivity and energy-based carrier scattering to increase the Seebeck coefficient.

Generally, MWCNTs have extraordinary mechanical stability and directional electrical transport; therefore, they are very attractive to embed as nanoinclusions in TE materials.^4,29−31^ CNTs themselves show superior TE performance due to their unique 1D holey geometry, excellent electronic transport, and low-dimensional nanoscale features.^30^ Moreover, MWCNTs are lightweight, are commercially available at low cost, have a negligible environmental impact, and are thermally and mechanically stable, flexible, and chemically inert.^29,31^ Although CNTs have ultrahigh thermal conductivity ∼600 W m^–1^ K^–1^, which did not contribute to composite thermal conductivity, since interfaces associated to large surface area help in strong high phonon scattering.^32^ In a similar study, Kim et al.^4^ added CNTs in the Bi_2_Te_3_ matrix, resulting in the reduction of thermal conductivity and enhancement of the Seebeck coefficient, which overall improved the TE performance. Khasimsaheb et al.^11^ added CNTs in the PbTe matrix and observed that the incorporation of the optimum amount of nanoinclusions resulted in a reduction in thermal conductivity and an enhancement in the power factor through an increase in both the Seebeck coefficient and electrical conductivity. Therefore, the added secondary phase’s size and distribution are crucial in electrical and thermal carrier transport mechanisms. The addition of an optimum concentration of nanoinclusions is necessary for fine dispersion, thermal carrier scattering, and electrical carrier filtering mechanisms.^29^ In this study, our strategy is to effectively composite Cu_3_SbS_4_ TE material with homogeneously dispersed MWCNT nanoinclusions to accentuate TE performance. We synergistically suppress the lattice thermal conductivity and improve the Seebeck coefficient through interfacial boundary scattering by forming the Cu_3_SbS_4_/MWCNT composite.

## Experimental Procedure

**Figure 1:**
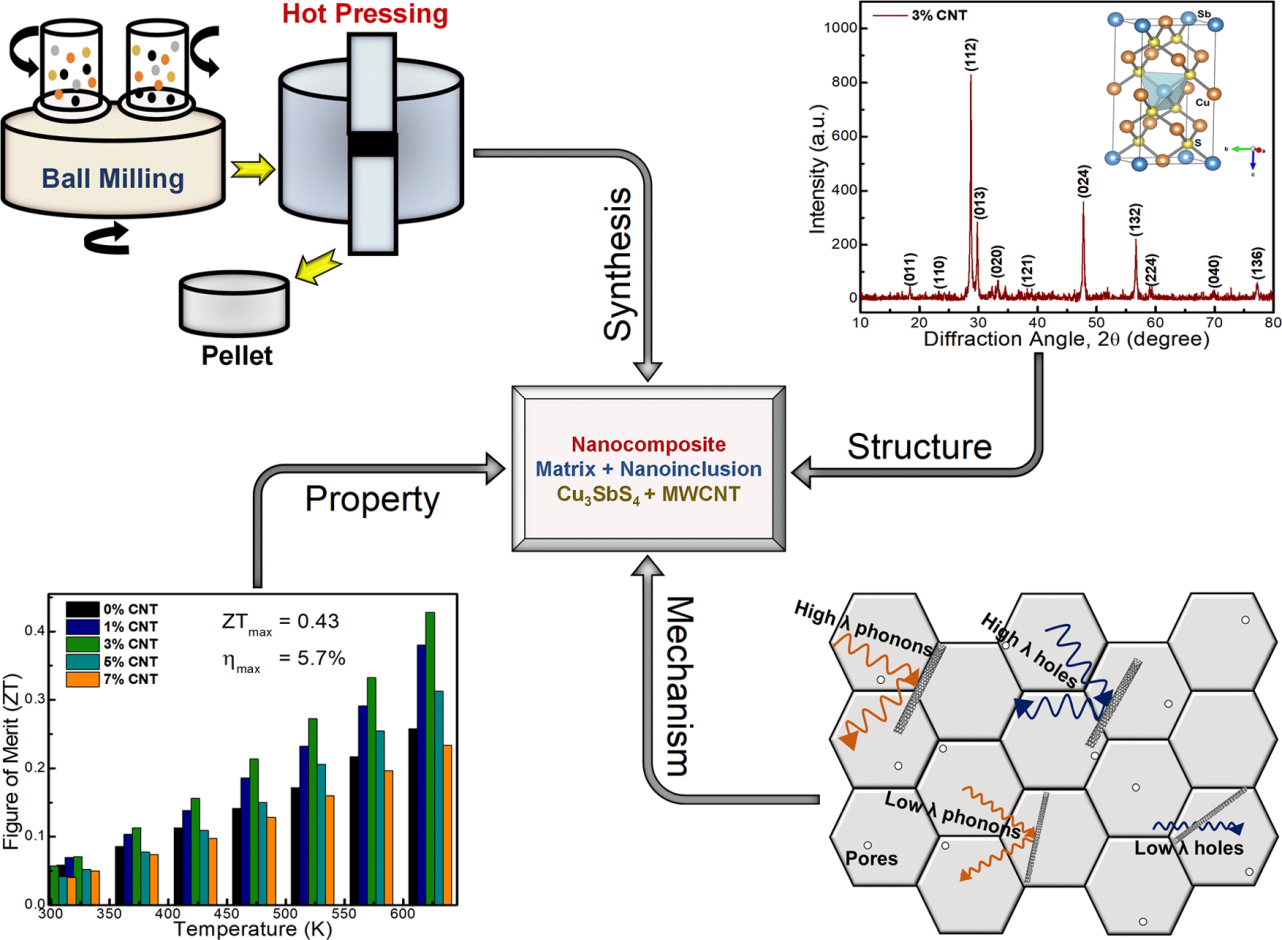
Schematic diagram showing the synthesis, structure, mechanism, and property relation of the designed nanocomposite (Cu_3_SbS_4_ + MWCNT) system.

Cu_3_SbS_4_-MWCNT composite materials were synthesized from the commercial elements of copper powder (Kurt. J. Lesker, purity: 99.9%), sulfur powder (Sigma Aldrich, purity: 99.98%), antimony pellets (Kurt. J. Lesker, purity: 99.999%), and multiwalled CNT with 8–15 nm diameter and up to 10 μm length (XFNano Inc.). Then, stoichiometric powders were crushed thoroughly using a pestle and an agate mortar to produce homogeneous mixtures. Various compositions of polycrystalline Cu_3_SbS_4_ + *x* atomic% of MWCNT (*x* = 0, 1, 3, 5, and 7) nanocomposites were synthesized by solid-state and facile two steps of the conventional mechanical alloying route, as shown in Figure 1. The nominal compositional powders were homogeneously mixed using a high-energy planetary ball mill with desired parameters (rotation speed: 300 rpm, duration: 24 h, ball-to-powder ratio: 25) using zirconia balls in a toluene medium. Then, the highly densified pellets were synthesized by compacting the ball-milled powders directly loaded into a steel mold with a 15 mm diameter die and hot-pressed at 400 °C under the uniaxial load of 120 MPa for 15 min. The standard Archimedes method was used to measure and calculate the experimental densities of the obtained pellets.

The formed crystalline phase study was investigated using the Bruker D2 Phaser instrument of powder X-ray diffraction method with a Lynxeye detector. For all samples, diffraction data were collected using Cu Kα radiation of wavelength 1.54 Å between the 2θ of 10 to 70° with a standard step size of 0.02°. Then, the standard Williamson–Hall equation was used to calculate the internal strain and crystalline size from the diffraction data. The Raman spectra were collected in WITec RAMAN alpha 300R equipment with an excitation DPL laser wavelength (cobalt) of 532 nm. To understand the distribution of carbon nanoinclusions, we also performed the Raman mapping of the G-band peak in the 1 μm × 1 μm square area. The ball-milled powders and MWCNT nanoinclusion microstructure were captured in the FESEM (model: Hitachi SU8240). The backscattered microstructure and elemental analysis were captured using the SEM (model: JEOL JSM IT500) with embedded compositional elemental mapping and energy-dispersive X-ray spectroscopy (EDX) to ascertain the MWCNT nanoinclusion distribution and remaining matrix elements.

From ambient temperature to 623 K, the electrical transport characteristics (Seebeck coefficient (*S*) and electrical conductivity (σ)) were measured using the conventional four-probe direct current measurement method using a Netzsch SBA 458 Nemesis measurement system. The Lakeshore Hall measurement system was employed to test the room-temperature charge carrier transport (carrier concentration and mobility) within the −2 to +2 T magnetic field range. Standard disk-shaped specimens approximately 12.7 mm in diameter and 2 mm in thickness were used to evaluate thermal diffusivity in Netzsch LFA 467 equipment using the laser flash technique in an inert environment. Netzsch-assisted software was used to assess the thermal conductivities using the standard formula κ = ρα*C*_p_, where ρ is the calculated density, *C*_p_ is the specific heat capacity, and α is the thermal diffusivity of the sample. The temperature-dependent Fermi level (*E*_f_), the room-temperature density of the states’ associated effective mass (*m*_d_^*^), and the effective density of states (eDOS) were assessed using the observed carrier concentration and the Seebeck coefficient in all of the samples. Mechanical stability (hardness) was evaluated using the micro-Vickers hardness tester on finely polished scratch-proof samples with a load of 5 kgf and a dwell time of 10 s.

## Results and Discussion

**Figure 2:**
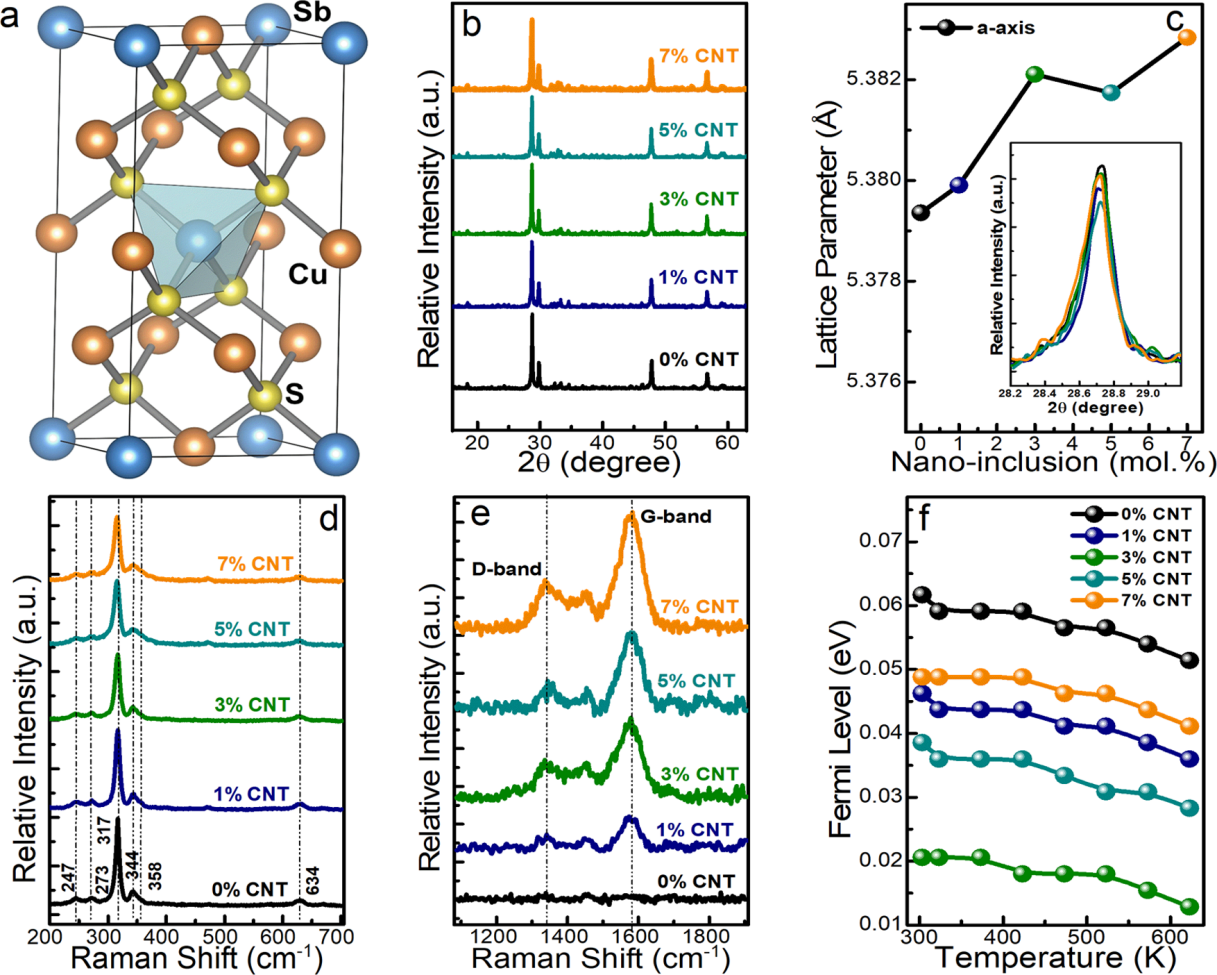
(a) Crystal structure, (b) XRD, (c) lattice parameter with inset showing XRD high-intensity peak shift, (d) Raman spectra of matrix, (e) Raman spectra of carbon peaks, and (f) Fermi level of Cu_3_SbS_4_ + *x*% MWCNT (*x* = 0, 1, 3, 5, and 7) nanoinclusion samples.

**Figure 3:**
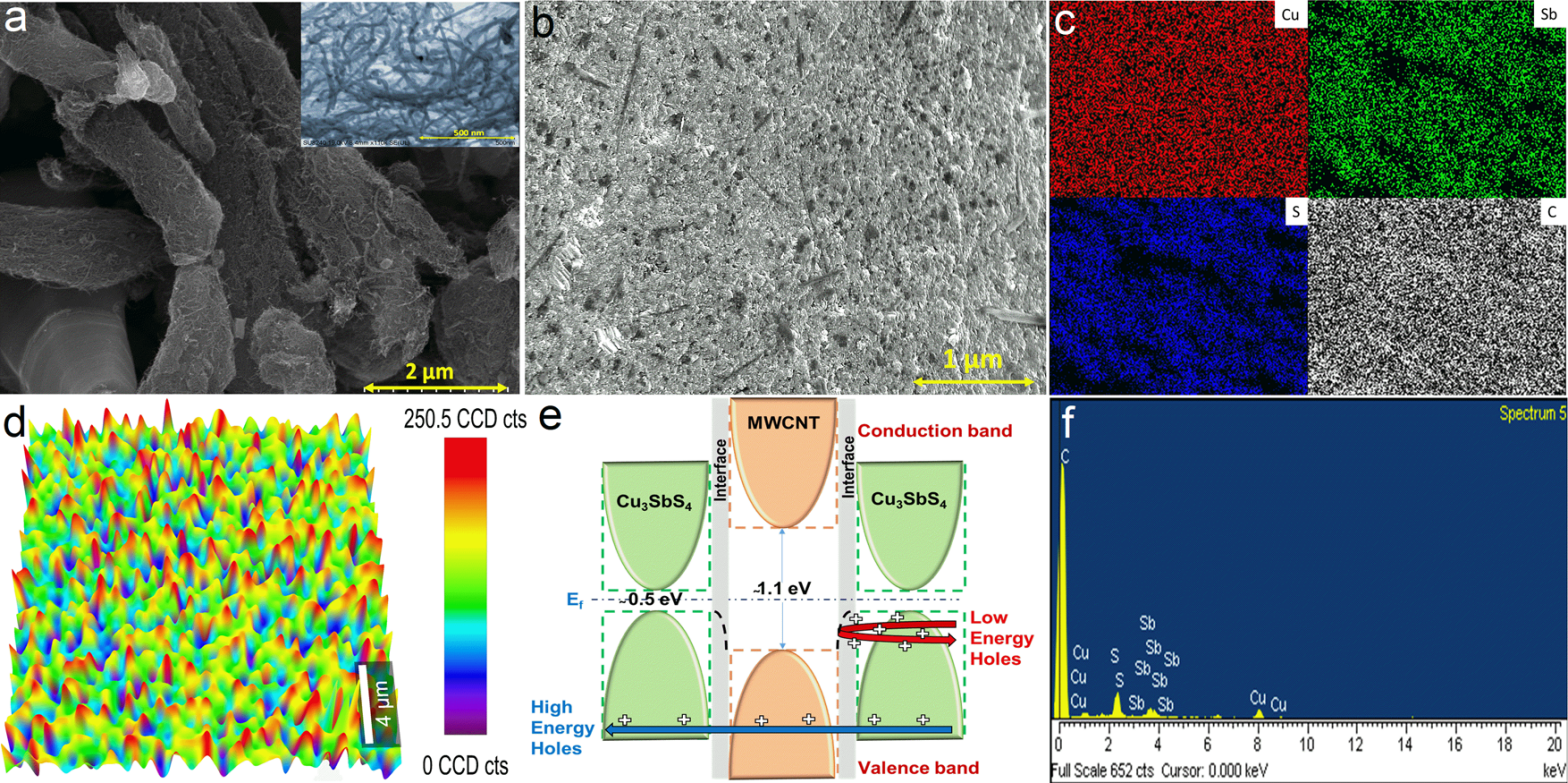
(a) FESEM micrograph of ball-milled powder with the inset showing added MWCNT, (b) SEM BSE micrograph, (c) elemental mapping, (d) Raman mapping, (e) energy filtering mechanism, and (f) EDX results of the Cu_3_SbS_4_ + MWCNT composite system.

The facile combination of ball milling and hot pressing is used to synthesize composites consisting of commercial MWCNT fillers and the Cu_3_SbS_4_ matrix, as shown in Figure 1. All X-ray diffraction (XRD) peaks perfectly match the Cu_3_SbS_4_ tetragonal structure without any extra peaks related to the inclusion phase or other secondary phases, as shown in Figure 2b. Added MWCNT concentration is very small to observe the carbon peaks using XRD analysis. The lattice parameter is not much altered (the increment is less than 0.06% between pristine and 7% loaded MWCNT samples) by the addition of nanoinclusions, and lattice parameter (*a*) is close to 5.38 in all of the samples, as shown in Figure 2c. We evaluated the crystalline size and internal microstrain using the Williamson–Hall reaction, and values are showcased in Table 1. The crystalline size (in nm) is reduced with the increasing concentration of nanoinclusions, which is due to the added inclusions not allowing the growth of grains during the sintering by pinning the grain boundaries. The internal microstrain increased with the increasing inclusion concentration because of the added MWCNT generating extra structural defects and boundaries in the lattice structure. As shown in Figure 2d, the Raman spectroscopy revealed that the peaks related to Cu_3_SbS_4_ exhibited vibration modes at 247, 273, 317, 344, 358, and 634 cm^*–*1^ Raman shifts.^33^ These Raman peak intensities are reduced in the composite samples due to the creation of a complex molecular environment by the added MWCNT nanoinclusions, crystalline size, and lattice defects. According to Figure 2e, carbon-based D- and G-band peaks are seen in nanocomposite samples, and their relative intensity increases as the concentration of MWCNT nanoinclusions increases. The microstructures of MWCNT nanoinclusions with added Cu_3_SbS_4_ ball-milled powder along with a clear contrast micrograph of the MWCNT nanoinclusions are captured in FESEM, as shown in Figure 3a. The morphology of the backscattered SEM microstructure revealed the homogeneous distribution of the MWCNT inclusions in the Cu_3_SbS_4_ composite samples (Figure 3b). The elemental mapping of the 3% MWCNT composite samples are shown in Figure 3c with the four major contributing elements of Cu, Sb, S, and C. SEM compositional mappings informs the homogeneous dispersion MWCNT in the matrix and even distribution of matrix elements without any secondary phases from the Cu–Sb–S combination. Raman mapping of the carbon-related G-band region of pristine and 3% MWCNT composite samples shows improved localized carbon intensity and homogeneous distribution of carbon in the composite sample (Figure 3d). The relative density is decreased with the increasing MWCNT concentration since increased boundary concentration generates back stress and stress concentration points near the interfaces against the densification.^34,35^ In Figure 1, we demonstrated the overall conceptual relation between synthesis, structural, mechanism, and TE performance of our designed Cu_3_SbS_4_–MWCNT composite system.

**Table 1:** Crystalline size, Relative Density, Fermi Level, Effective Mass, Effective DOS, and Internal Strain of the Cu_3_SbS_4_ + *x*% MWCNT Nanoinclusion Samples (*x* = 0, 1, 3, 5, and 7)

inclusion (mol %)	crystalline size (nm)	relative density (*%*)	Fermi level (meV)	effective mass (m_d_^*^)	effective DOS (×10^20^ cm^3^)	internal strain (×10^–4^)
0	42	97.4	61.68	0.12	0.01	5.58
1	37	95.9	46.26	0.65	0.13	6.13
3	38	95.2	20.56	1.03	0.27	6.68
5	37	94.9	38.55	1.59	0.51	8.53
7	36	94.8	48.83	2.87	1.24	8.20

**Figure 4:**
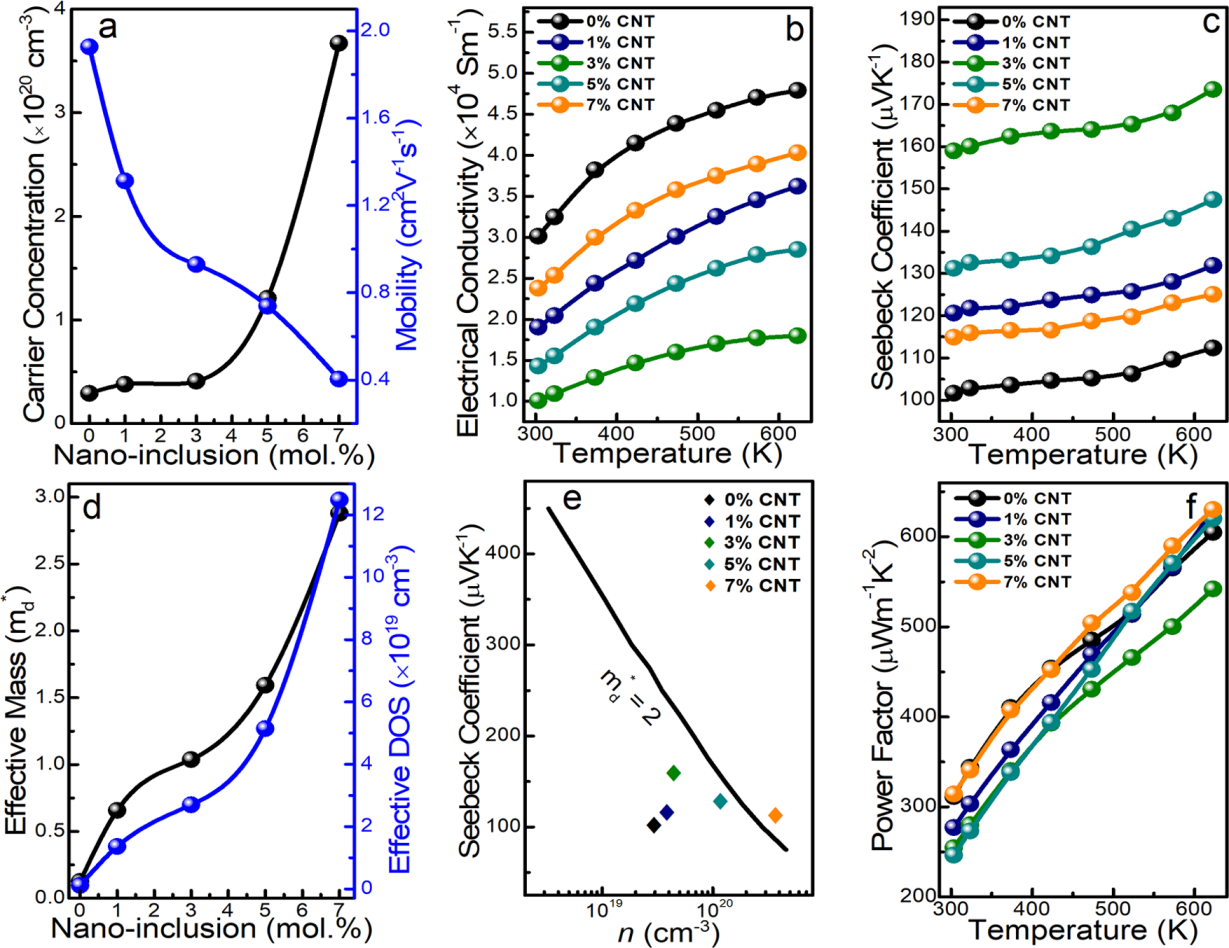
Charge carrier transport properties of (a) carrier concentration and mobility, (b) electrical conductivity, (c) Seebeck coefficient, (d) room-temperature density of states effective mass and effective DOS, (e) Pisarenko relation, and (f) power factor of Cu_3_SbS_4_ + *x*% MWCNT (*x* = 0, 1, 3, 5, and 7) nanocomposite samples.

Hall measurement at room temperature revealed the carrier concentration in the order of 10^20^ cm^*–*3^, which increases with increasing the loadings of MWCNT nanoinclusions, as shown in Figure 4a. As shown in Figure 4a, mobility is reduced by increasing the MWCNT inclusions due to the matrix carriers scattering by the nanoinclusion-associated interfacial boundaries. According to Figure 4b, electrical conductivity increases with temperature, demonstrating that all samples exhibit degenerated semiconductor behavior. The formation of the new thermally generated electron–hole pairs with increasing temperature improves the electrical conductivity.^36^ However, as shown in Figure 4b, the overall electrical conductivity was reduced by adding MWCNT inclusions, suggesting the dominance of charge carrier scattering (i.e., decreased mobility) by interfacial boundaries.^37^ The reduced carrier mobility dominated the improved carrier concentration added by the MWCNT nanoinclusions to the overall electrical conductivity. The increased porosity level with the increasing MWCNT concentration also acts as an obstacle for electrical transport.^38^ Toward this end, Kim et al.^4^ observed similar electrical conductivity reduction with the inclusions of MWCNT in bismuth telluride, and the reason mentioned was newly formed heterostructure interfaces. However, above the 3% MWCNT concentration, the electrical conductivity increases but not over that of the pristine sample, as shown in Figure 4b. Increasing the nanoinclusion concentration above 3%, the high-aspect-ratio nanotubes begin to form clusters or close together to generate the percolation effect for improved charge carrier transport.^36^ Theoretically, the calculated Fermi level was obtained through experimental data from eqs 1 and 2.
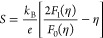
1
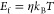
2

Fermi level (*E*_f_) was almost unchanged with temperature and reduced gradually with the increasing concentration of MWCNT inclusions, as shown in Figure 2f. Effective mass and effective density of states are also calculated theoretically using the single parabolic (SPB) model from experimental data using eq 3 (Mott’s formula) and eq 4. As shown in Figure 4d, as the concentration of MWCNT nanoinclusions increases, the corresponding density of states effective mass and effective density of states gradually increased.
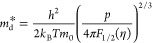
3
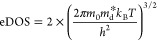
4

As shown in Figure 4c, the Seebeck coefficient increased with the increasing temperature in all the samples. The measured Seebeck coefficient data in all of the samples show positive values throughout the temperature range, indicating assertive p-type semiconductive behavior. Interfacial boundaries generated by MWCNT nanoinclusions filter the energy-dependent charge carriers based on the band alignment and band gap difference between the matrix and inclusion phase. Gayner et al.^8^ demonstrated that the Seebeck coefficient improves if the low-energy charge carriers scatter, negatively contributing to the overall normalized Seebeck distribution. The mechanism is known as carrier energy filtering, which is one of the common phenomena in composite structures, where the interfacial boundaries filter low-energy carriers, as shown in Figure 3e. Above the 3% MWCNT inclusion concentration, a reduction in the Seebeck coefficient was observed due to the increased charge carrier concentration contribution, dominating the energy filtering mechanism. As shown in Figure 4e, the Pisarenko relation based on the single parabolic model between experimentally measured Seebeck coefficient and carrier concentration satisfies the energy filtering effect up to 3% MWCNT concentration. As shown in Figure 4f, the increase in the calculated power factor is almost unchanged by adding MWCNT inclusions, especially at high temperatures.

**Figure 5:**
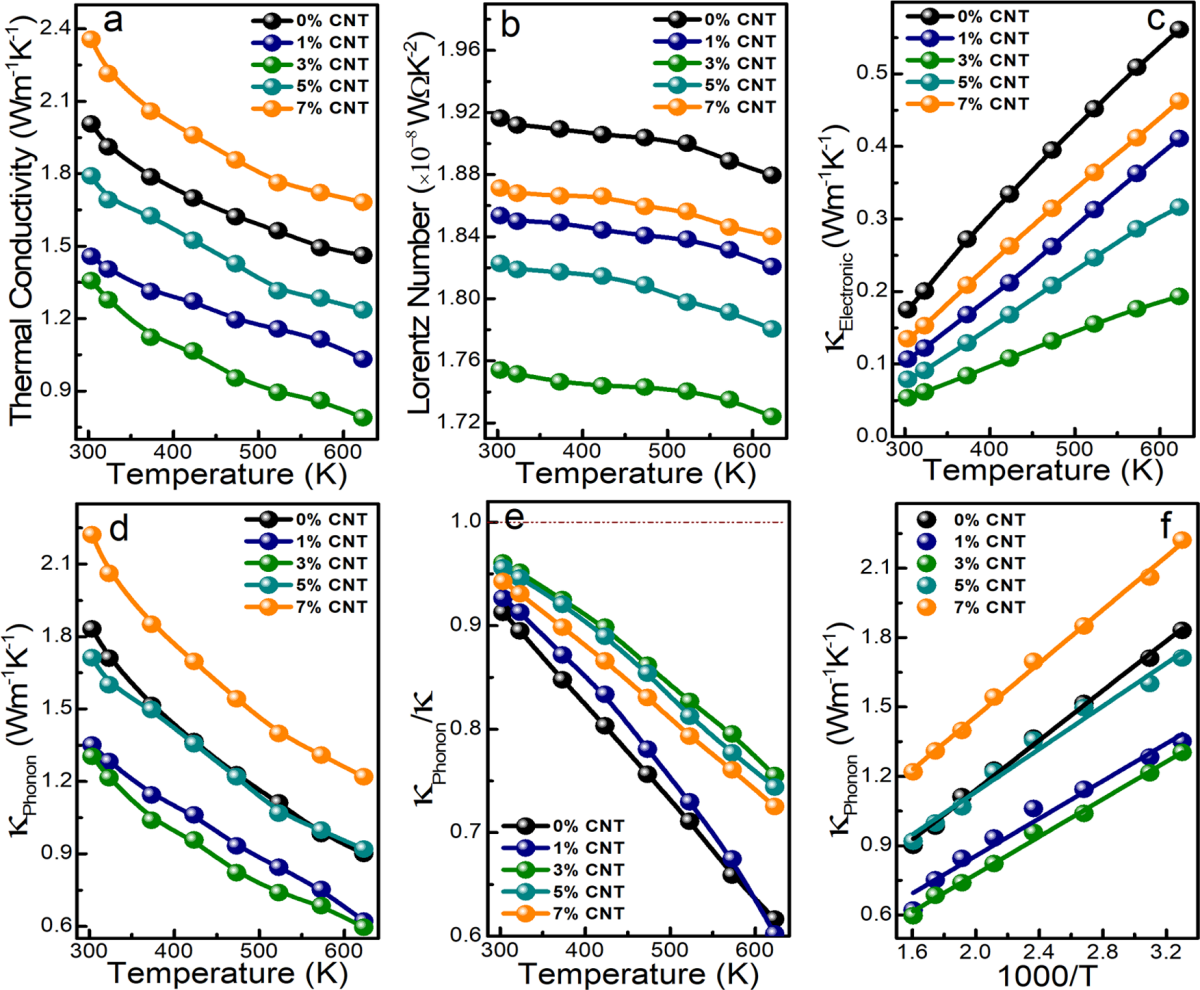
Thermal carrier transport properties of (a) total thermal conductivity, (b) Lorentz number, (c) electronic part of the thermal conductivity, (d) phonon part of the thermal conductivity, (e) phonon contribution on total thermal conductivity, and (f) κ_Phonon_ versus 1000/*T* of Cu_3_SbS_4_ + *x*% MWCNT (*x* = 0, 1, 3, 5, and 7) nanocomposite samples.

As shown in Figure 5a, with the increasing temperature, a gradual decrease in total thermal conductivity is observed in all of the samples. The lowest thermal conductivity of 0.8 W m^*–*1^ K^*–*1^ was noticed in the 3% MWCNT nanoinclusion sample, which is almost half of the pristine sample at 623 K. The addition of MWCNT inclusions successfully scattered the phonons through heterostructure interfacial boundary phonon scattering. The thermal conductivity of Cu_3_SbS_4_ is reduced successfully by forming a nanocomposite structure up to the addition of the optimized amount of 3% MWCNT nanoinclusions. However, above the 3% loadings of MWCNT, the thermal conductivity was increased, which is attributed to the contributions of the high thermal conductivity of MWCNT nanoinclusions. The percolation effect discussed for electrical conductivity also improves thermal conductivity above the 3% MWCNT concentration. To understand individual thermal conductivity contribution from electronic (κ_e_) and phonon (κ_p_) parts, we used the Wiedemann–Franz law, i.e., κ_e_*= L*σ*T* and total thermal conductivity κ = κ_e_*+* κ_p_, where σ is electrical conductivity, *L* is the Lorenz number, and *T* is the absolute temperature. In a similar work, Kim et al.^39^ demonstrated a standard equation to evaluate the Lorenz number from the experimental Seebeck coefficient, that is *L* = 1.5 + exp(−|*S*|/116), where *S* has units of μV/*K* and *L* of 10^*–*8^ WΩ K^–2^, as shown in Figure 5b. As depicted in Figure 5c, κ_e_ is reduced by MWCNT nanoinclusions and slowly starts increasing with increasing the concentration of MWCNT by more than 3%. In all of the samples, κ_e_ increases with increasing temperature due to the contribution of the excited minority carriers at higher temperatures. In all of the samples, the phonon part of the thermal conductivity shows a similar trend to total thermal conductivity (Figure 5d). The phonons are major thermal carriers and contribute more than electrons toward total thermal conductivity, as shown in Figure 5e. To understand the primary mechanism associated with the phonon thermal conductivity, we plotted κ_p_ over 1000/*T* (Figure 5f). The solid linear relationship between κ_p_ and 1000/*T* with the negative slope with the increasing temperature indicates the dominance of the Umklapp phonon–phonon scattering mechanism in all of the samples.^40^

**Figure 6:**
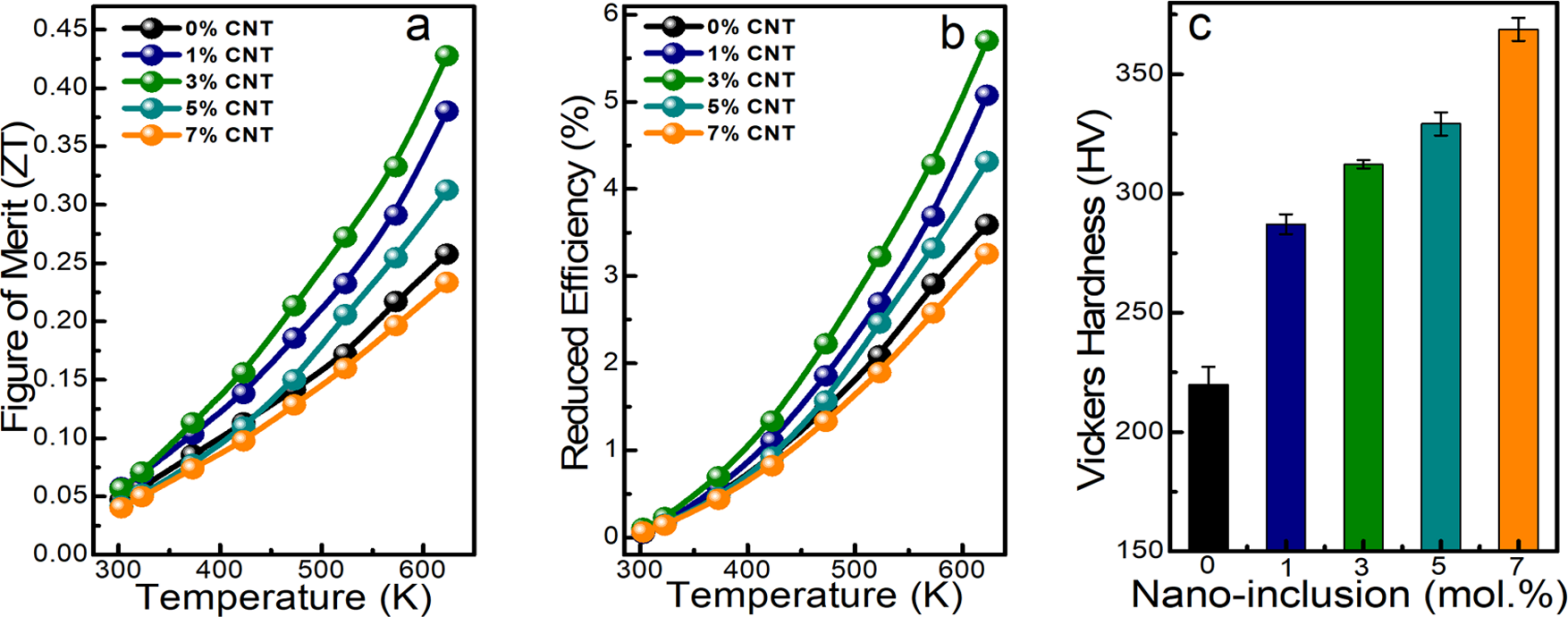
(a) Figure of merit, (b) reduced efficiency, and (c) Vickers hardness of Cu_3_SbS_4_ + *x*% MWCNT (*x* = 0, 1, 3, 5, and 7) nanocomposite samples.

As presented in Figure 6a, we calculated the figure of merit in all of the samples and witnessed a maximum *ZT* in the 3% MWCNT nanocomposite sample at 623 K. The maximum *ZT* of 0.43 achieved in the 3% MWCNT sample is almost 70% higher than that of the pristine Cu_3_SbS_4_ with *ZT* of 0.25 at 623 K. However, above 3% MWCNT concentration, *ZT* is reduced due to increased thermal conductivity from MWCNT nanoinclusions. Therefore, 3% MWCNT is the optimum homogeneously distributed nanoinclusions for attaining maximum thermoelectric performance in Cu_3_SbS_4_. As shown in Figure 6b, the reduced efficiency of the materials is calculated from the obtained *ZT* using eq 4.
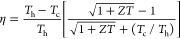
5

We attained a reduced efficiency of 3.7% in the pristine sample and maximum reduced efficiency of 5.7% in the 3% MWCNT loaded sample. The mechanical stability (Vickers microhardness) is calculated in all of the samples and presented in Figure 6c. Vickers hardness is gradually increased with the concentration of nanoinclusions from 220 HV in the pristine Cu_3_SbS_4_ to 370 HV in the 7% MWCNT nanocomposite sample. We strongly believe that the incorporated intrinsically stable, high-strength MWCNT inclusions generate dispersion hardening, and interfacial and fine grain boundaries induce boundary hardening to improve the mechanical stability of the composite samples. Cu_3_SbS_4_/MWCNT composites represent an improved TE performance and mechanically robust material for promising next-generation TE material for intermediate-temperature applications.

## Conclusions

In summary, Cu_3_SbS_4_ + *x*% MWCNT (*x* = 0, 1, 3, 5, and 7) nanocomposite samples are successfully synthesized by employing facile ball milling, followed by a hot pressing route. The incorporation of MWCNT nanoinclusion generates heterostructure interfaces, fine grain boundaries, and structural lattice defects that help form thermal carrier barriers for scattering phonon transport. In addition, added MWCNT nanoinclusions scatter the low-energy charge carriers through carrier energy filtering, which enhanced the Seebeck coefficient. Therefore, the power factor is maintained constant; despite, the electrical conductivity is reduced. Adding the optimized amount of MWCNT nanoinclusions to Cu_3_SbS_4_ improved the overall thermoelectric performance and the reduced conversion efficiency. An increased figure of merit of 0.43 and a reduced efficiency of 5.7% is witnessed for the 3 mol % MWCNT sample at 623 K. Furthermore, the addition of MWCNT nanoinclusions improved mechanical stability (hardness) and exhibited a proportional relation with nanoinclusion concentration. Two mechanisms of dispersion strengthening and grain boundary hardening helped increase the hardness from 220 HV in the pristine sample to 370 HV in the 7% MWCNT sample. Our study strongly believes that Cu–Sb–S-based materials compositing with MWCNT nanoinclusions is an efficient, nontoxic, inexpensive, and mechanically stable route for next-generation thermoelectric devices for intermediate applications.
